# Deficits in multiple object-tracking and visual attention following mild traumatic brain injury

**DOI:** 10.1038/s41598-022-18163-2

**Published:** 2022-08-12

**Authors:** Mohammed M Alnawmasi, Sieu K. Khuu

**Affiliations:** 1 School of Optometry and Vision Science, The University of New South Wales, Sydney, Australia; 2 College of Applied Medical Science, Department of Optometry, Qassim University, Buraydah, Saudi Arabia

**Keywords:** Eye diseases, Neurological disorders, Trauma

## Abstract

Difficulty in the ability to allocate and maintain visual attention is frequently reported by patients with traumatic brain injury (TBI). In the present study, we used a multiple object tracking (MOT) task to investigate the degree to which TBI affects the allocation and maintenance of visual attention to multiple moving targets. Fifteen adults with mild TBI and 20 control participants took part in this study. All participants were matched for age, gender, and IQ. The sensitivity and time taken to perform the MOT task were measured for different conditions in which the duration of the tracking, number of target, and distractor dots were systematically varied. When the number of target dots required to be tracked increased, sensitivity in correctly detecting them decreased for both groups but was significantly greater for patients with mild TBI. Similarly, increasing the number of distractor dots had a greater effect on reducing task sensitivity for patients with mild TBI than control participants. Finally, across all conditions, poorer detection performance was observed for patients with mild TBI when the tracking duration was longer compared to control participants. The present study showed that patients with mild TBI have greater deficits (compared to control participants) in their ability to maintain visual attention on tracking multiple moving objects, which was particularly hindered by increased tracking load and distraction.

## Introduction

Traumatic brain injury (TBI) typically occurs when an external mechanical force causes diffuse or localised damage to neural structures in the brain^1,2^. It is a common neurological disorder with annual reports of approximately 2.87 million TBI- related emergency visits in the U.S. due to falls, struck by or against an object, and motor vehicle accidents^3^. TBI is typically categorised as being mild, moderate and severe based on conventional scaling systems such as the Glasgow Coma Scale (GCS) and the duration of post-traumatic amnesia (PTA)^1,4^.

Of the annually reported cases of TBI, almost 90% of TBI are mild and is often interchangeably referred to as ‘concussion’ and usually characterised by a change to normal brain function for no longer than a few minutes following trauma^4^. Mild TBI is usually characterised by diffuse pathophysiological brain damage (which may not be detectable in routine diagnostic brain imaging), particularly to axonal fibres, which may be damaged from sheering and acceleration/deceleration forces^5–7^. The effect of mild TBI can be immediate, such as a loss of consciousness or confusion, or may persist long-term disturb physical, emotional, cognitive, and visual abilities^8,9^.

Mild TBI is known to be associated with a variety of symptoms which can negatively influence daily living and a myriad of behaviors^10,11^. These symptoms may be somatic (e.g., nausea, dizziness, headaches, difficulty with balance, confusion, and disorientation), cognitive (memory, attention, poor concentration, and processing speed), and vision related symptoms (light sensitivity, visual field deficits, accommodation, and eye movement abnormalities)^10,12,13^. In general, TBI related symptoms typically resolve within 1 to 3 months post injury, however, some symptoms may persist for weeks, months or even years^14^. Persistent and long-term symptoms from mild TBI is commonly referred to as post-concussion syndrome^13^.

Previous studies have widely reported that visual disturbances are among the more common mild TBI related symptoms and can affect everyday visually dependent tasks, such as reading, searching, and driving^15^. These tasks require not only clear and sharp vision but also cognitive functions such as attention to focus on visual information for guided behaviour and decision making^16,17^. Typically, visual attention can be characterized based on different ‘components’ which may reflect different strategies or means through which attention is allocated and utilized to process information. Visual attention can be selective, which refers to the ability to direct and focus on specific visual information whilst ignoring irrelevant information^18^. Visual attention can be divided and refers to the ability to attend to more than one stimulus at the same time^19^. Finally, visual attention can be sustained and relates to the ability to maintain attention to a specific visual stimulus/stimulus over time without fluctuation in performance^20,21^.

Specific deficits in selective visual attention has been reported following TBI^22–25^, attention allocation^26–29^, divided visual attention^22^, and sustained visual attention have been widely reported in the literature^30–32^. In contrast, other studies have failed to find evidence for visual attentional deficits following TBI^33–35^. However, differences in outcomes may be driven by methodological approaches and the severity of TBI (see Alnawmasi et al., 2022 for a review), and more research is needed to fully characterise the extent to which visual attention is affected by TBI^36^.

In characterising deficits visual deficits following mild TBI, the majority of studies have used tasks and procedures that have investigated the initial ‘allocation’ of attention and minimum time required for participants to detect and respond to the presence (or absence) of a target stimulus. Typically, variants of the visual search paradigm are employed in which the time required to ‘allocate’ attention is measured^37,38^. Whilst such studies have been informative in revealing deficits in the speed of processing from TBI, they do not inform about potential deficits in other attentional processes, such as sustaining attention over an extended continuous period. This is not a limitation of previous approaches in measuring attention, or the methods adopted to investigate attentional processes, but rather have been largely focussed on one component of visual attention. Thus, there are limitations in our understanding of the full impact of TBI on general cognitive function. As noted, a handful of studies have measured ‘sustained’ visual attention, but this is done in the context of investigating how the ability to initially allocate attention changes over time (by repeatedly measuring visual search at fixed intervals over time), and not the degree to which visual attention can be continuously maintained when engaged in a task. Maintaining attention is a major feature of common daily behaviours, such as driving, reading, and concentration, and it is unclear whether it is affected following TBI. The goal of the present study was to investigate this issue using a multiple object tracking paradigm in which we quantified the ability to allocate and maintain visual attention on multiple objects that move for an extended period of time.

**Figure 1:**
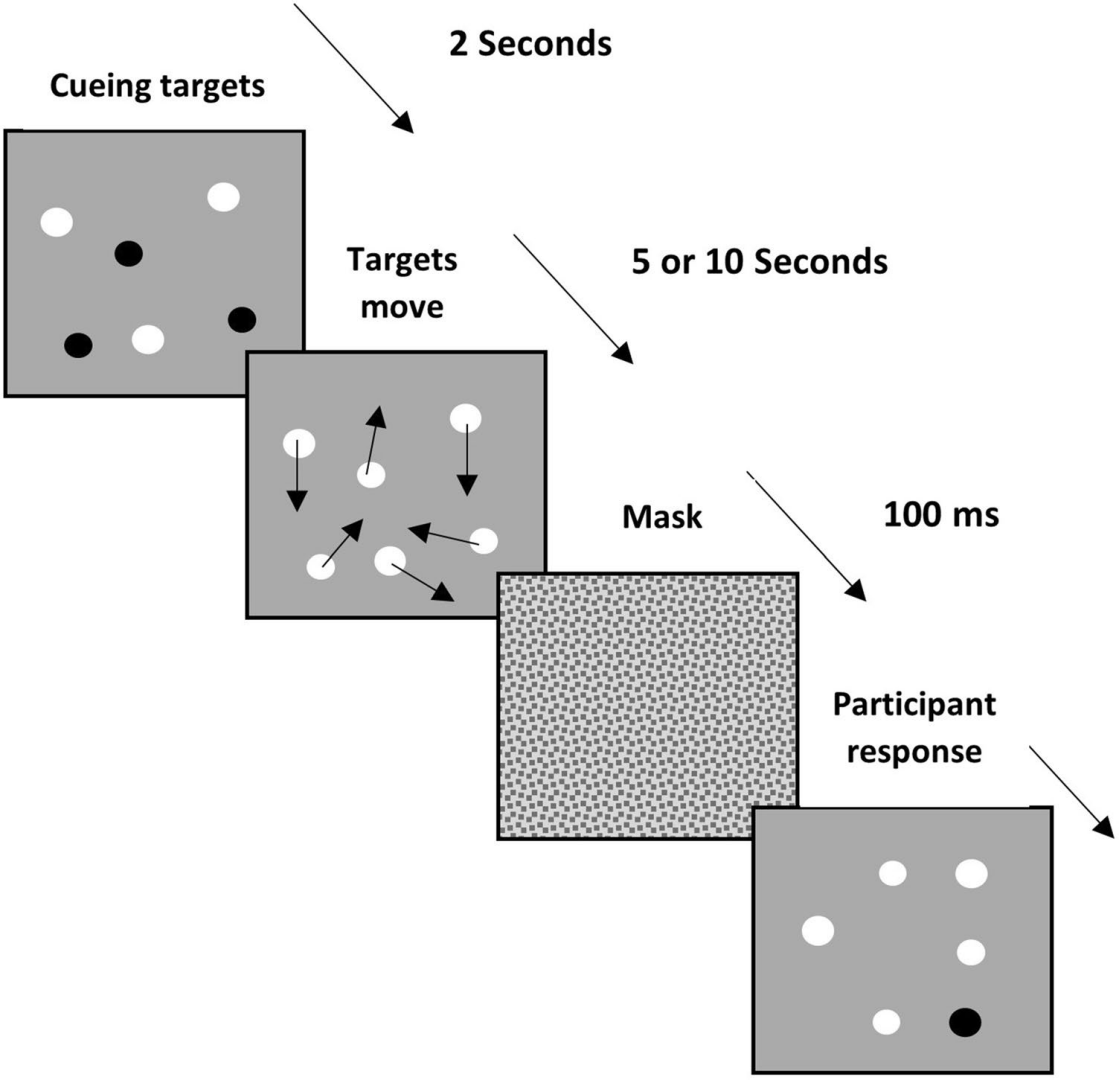
A schematic diagram of multiple objects tracking task used in the present study. Initially, target dots were cued and after a brief delay period, all dots moved randomly for a fixed period of time of 5 or 10 s. At the end of this period, a mask was quickly shown before one of the dots was cued and the participants were required to judge whether it was a target dot.

The multiple object tracking (MOT) paradigm and task was first developed by Pylyshyn and Storm^39^. Typically, the MOT task comprises a fixed number of identical elements (such as circular dots) presented on a computer screen. Elements can be target or distractors. Initially, all elements are stationary, and the locations of a number of target objects are cued (typically by briefly flashing them); objects that are not cued act as distractors. All elements undergo random motion, and participants are required to maintain attention on the cued objects for a fixed period of time. At the end of the trial, all elements stop moving and a randomly selected single element is cued, and the participant is required to indicate whether it was a target element (see Fig. 1). Pylyshyn and Storm reported that participants, on average, were able to track up to 5 objects among a similar number of distractors. However, performance is highly dependent on the tracking duration and the number of distractor objects^40,41^.

Several models have been proposed to explain the mechanism underling the ability of tracking multiple moving objects^40,42^. According to Yantis, multiple tracking is synonymous with perceptual grouping, and during tracking the visual system perceptually groups object into a single global object based on Gestalt grouping principles^43^. Alternatively, the Serial Attentional Switching Model proposes that tracking ability is achieved by rapid serial-switching of attention between the locations of the objects that are to be tracked^40^, and no grouping is required. Note that both the perceptual grouping and Serial Attentional Switching Model approaches posit that attention is singularly focused on either the global or a local stimulus location, respectively. However, recently, researchers have proposed that attention can be multifocal and in which numerous objects can be attended to at once^44,45^. This model posits that, during multiple objects tracking, observers can split their attention between different target locations and suppress information at unattended locations^46,47^.

The utility of the MOT task as a measure of sustained and attention maintenance has been demonstrated in numerous studies investigating normal functioning such as driving^48^ and in neurological disorders such as schizophrenia and autism spectrum disorder^49,50^. For example, Kelemen et al. investigated attentional tracking using a MOT stimulus in 30 patients with schizophrenia and matched healthy control participants^49^. In their task, the participants were required to track 4 rectangles among 4 similarly shaped distractors. They found that patients with schizophrenia perform poorly compared with normal participants. Interestingly, they noted that spatial working memory ability can predict the performance on the MOT task. Similar patterns of impaired MOT performance have been reported in other neurodevelopmental disorders such as autism, which suggests that deficits in the ability to maintained attention may be a common morbidity in the abnormal brain^50^.

Given previous reports of deficits in selective and divided attention among patients with TBI^16,51,52^, which are potentially important to multiple motion tracking, our hypothesis is that MOT performance is affected following TBI. It was our goal to confirm this and establish the extent of deficit by comparing MOT performance between patients with TBI and normal control participants on conditions in which the number of target and distractor dots were systematically varied as well as the tracking duration. These conditions allowed us to investigate whether any potential deficits in MOT (following TBI) are observed when attention is selectively divided to track increasingly more target dots, and the susceptibility of attentional tracking due to noise (from increasing distractors). In addition to comparisons between control participants and patients with mild TBI, we also performed correlation analysis to explore whether performance on MOT is associated with TBI symptom scores acquired through the administration of 3 different standardized questionnaires that measure general, visual and attention related symptoms.

## Results

### Demographics

**Figure 2:**
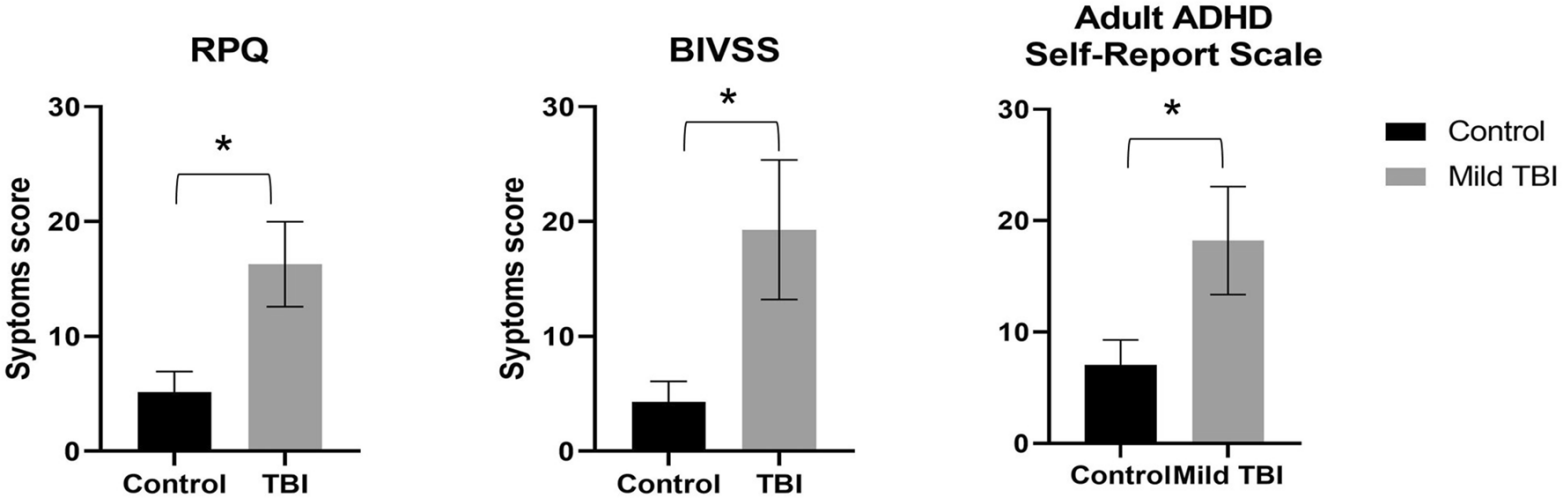
Mean symptoms scores for Rivermead post-concussion symptoms questionnaire (RPQ), brain injury vision symptom survey (BIVSS) questionnaire, and The Adult ADHD Self-report Scale. Error bars represent the standard error of the mean (± SEM). *p < 0.05.

The demographics for mild TBI and control groups are reported in Table 1. The two groups did not differ in terms of age, gender, and IQ (p < 0.05). Injury severity characteristics of patients with mild TBI are reported in Table 2. The major cause of mild TBI was motor vehicle accidents (n = 13, 87%), from a fall in one case, and sports-related injuries in one case. Patients with mild TBI were all chronic, in which they had TBI symptoms longer than at least 3 months after the initial date of injury. Time since injury ranged from 3 to 38 months. Patients with mild TBI reported significantly higher scores for general, visual and attention related symptoms as shown on Fig. 2. First, the RPQ scores ranged from 5 to 43 (mean 17.9 ± 14.5) for the 15 patients with mild TBI and ranged from 0 to 21 (mean 5.1 ± 6.7) for the 20 control participants (U = 42.5, p = 0.005). Secondly, BIVSS scores ranged from 0 to 60 (mean 21 ± 22.9) for the 15 patients with mild TBI and ranged from 0 to 7 (mean 2.7 ± 2.6) for the 20 control participants (U = 41, p = 0.01). Finally, Adult ADHD scores ranged from 2 to 52 (mean 19.3 ± 18.2) for the 15 patients with mild TBI and ranged from 0 to 26 (mean 7.1 ± 8.3) for the 20 control participants (U = 53, p = 0.02).

**Table 1:** Demographic characteristics for the control and mild TBI groups—M (SD).

	Mild TBI mean (SD)	Control mean (SD)
Gender ratio M/F	11/4	13/7
Age	31 (4.9)	29 (4.2)
IQ scores (using WASI)	91.8 (9.1)	93.5 (11.1)
RPQ*	17.4 (14.4)	3.8 (3.9)
BIVSS*	20.5 (24.7)	3.5 (2.8)
Adult ADHD Self-report Scale*	22.87 (20.5)	7.11 (7.3)
Initial GCS	14.6 (0.49)	NA
Duration of LOC (mins)	15.2 (58.1)	NA
Post injury period (months)	23.9 (26.7)	NA

*WASI-II* The Wechsler Intelligence Scale, *RPQ* The Rivermead Post-Concussion Symptoms Questionnaire, *BIVSS* Brain Injury Vision Symptom Survey, *GCS* Glasgow Coma Scale, *LOC* loss of consciousness; *Indicates a significant difference between patients with mild TBI and controls (at an alpha of 0.05).

**Table 2:** Injury characteristics of patients with mild TBI.

Participant #	Gender	Age	PIP (days)	Injury aetiology	Injury severity indices			Severity
					GCS	LOC	PTA	
A01	F	27	690	Motor vehicle accident	NA	0	0	Mild
A02	F	29	792	Motor vehicle accident	15	2 min	2 h	Mild
A03	M	41	99	Fall	14	15 min	0	Mild
A04	M	25	135	Motor vehicle accident	NA	10 min	NA	Mild
A05	F	37	720	Motor vehicle accident	NA	30 min	NA	Mild
A06	M	34	303	Motor vehicle accident	15	5 min	0	Mild
A07	M	23	396	Motor vehicle accident	15	0	0	Mild
A08	M	34	105	Football related injury	NA	20 min	NA	Mild
A09	M	31	270	Motor vehicle accident	NA	4 h*	24 h	Mild
A10	M	32	2880	Motor vehicle accident	NA	10 min	0	Mild
A11	M	37	2160	Motor vehicle accident	NA	30 min	27 h	Mild
A12	F	27	90	Motor vehicle accident	14	60 min	NA	Mild
A13	M	31	600	Motor vehicle accident	NA	5 min	0	Mild
A14	M	28	95	Motor vehicle accident	NA	30 min	24 h	Mild
A15	M	30	1440	Motor vehicle accident	15	2 min	0	Mild

*PIP* Post-injury period, *GCS* Glasgow Coma Scale, *LOC* duration of loss of consciousness, *PTA* duration of post-traumatic amnesia, *min* minutes, *NA* no data available. *Normally LOC duration is less than 30 min, but the diagnosis of TBI for this patient was made by a physician.

### Multiple object tracking task

Performance on the multiple objects tracking tasks was analyzed separately for sensitivity and reaction time outcome measures.

#### Sensitivity

**Figure 3:**
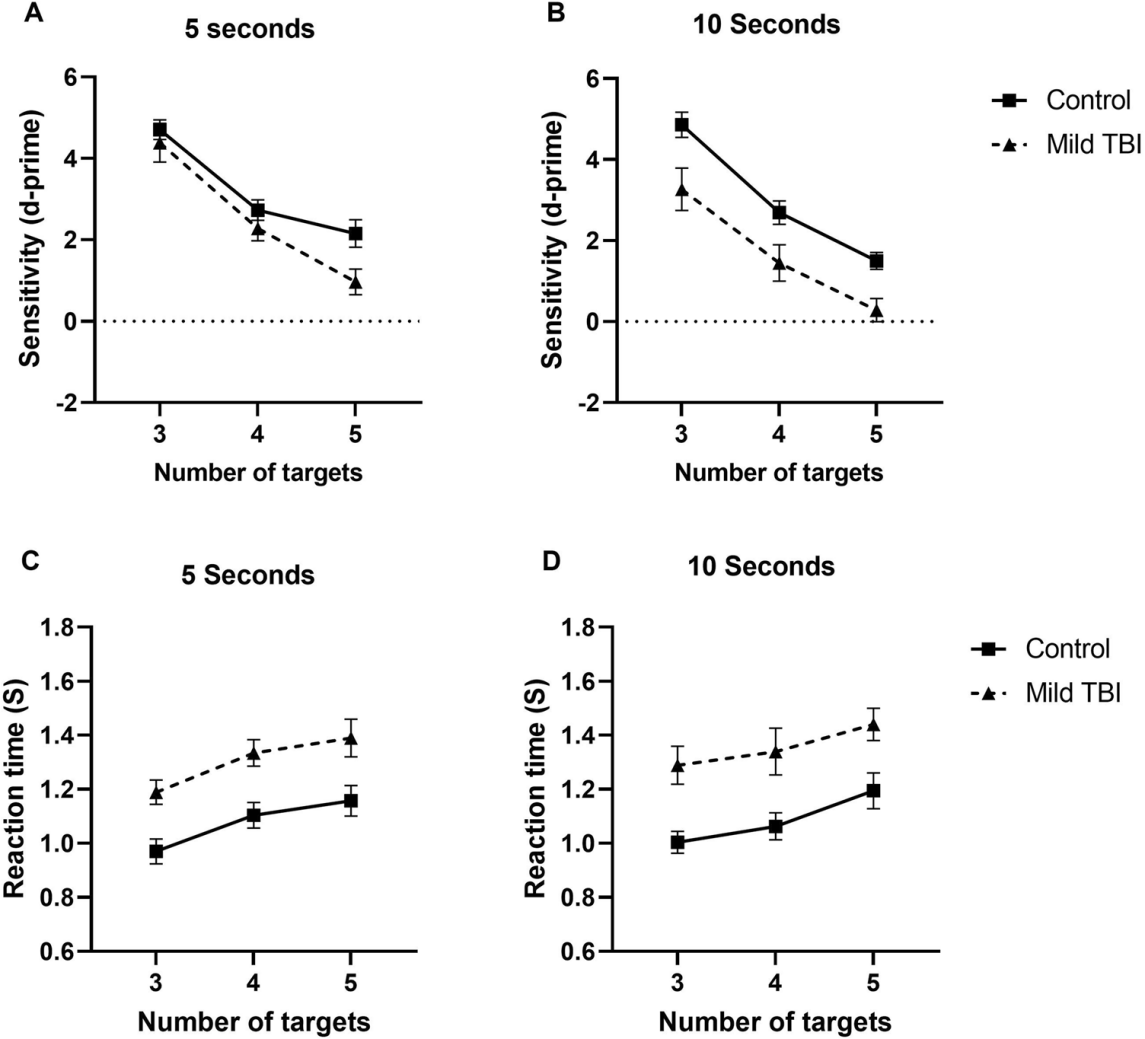
The sensitivity (**A**,**B**), and reaction time RT (**C**,**D**) as function of the number of targets to be tracked for control participants and patients with mild TBI (different symbols) for the tracking duration of 5 s (left panel) and 10 s (right panel). Error bars indicate 1 standard error of the mean.

Figure 3A and B plots sensitivity index (group average d-prime) as a function of the number of target dots (3, 4 and 5) for patients with mild TBI and control participants (different symbols) and different tracking durations (5 and 10 s—different panels). D-prime values are indicative of task performance (taking into consideration both hits and false alarm judgements) and higher values indicate greater discriminability and accuracy in judging whether the cued dot at the end of a trial was a target. Note also that in conditions in which there was a small target number of 3, d-prime values are high, indicating extreme high discriminability.

D’Agostino-Pearson test revealed that the data for all conditions and group were normally distributed (p = 0.05). As shown in these figures, as the number of target dots increased, there was a decrease in sensitivity in discriminating the target dots. In addition, overall, performance was poorer for patients with mild TBI compared to control participants, though this effect was dependent on the duration. We conducted a two-way ANOVA separately for durations of 5 and 10 s to determine whether group (TBI vs normal) and target dot number affected sensitivity. For a duration of 5 s, we find a main effect of both group (F (1, 96) = 6.032, p = 0.01, η^2^_p_ = 0.06) and target dot number (F (2, 96) = 43.78, p < 0.0001, η^2^_p_ = 0.48), but no significant interaction effect (F (2, 96) = 1.003, p = 0.37, η^2^_p_ = 0.02). While the effect size for group difference is small, as shown in Fig. 3, this is clearly dependent on the number of elements to be tracked. Indeed, Sidak, post-hoc analysis comparing performance between the two groups, showed that there was only a significant difference in group at the largest target dot number of 5 (3 target dots: MD = 0.32, p > 0.99; 4 target dots: MD = 0.45, p = 0.97; 5 target dots: MD = 1.19, p = 0.008). Additionally, Cohen’s d effect sizes (indicating the magnitude of difference between patients with mild TBI and control participants) progressively increased with the number of target dots (3 target dots: ES = 0.22, 95% CI [− 0.45, 0.89]; 4 target dots: ES = 0.57, 95% CI [− 0.11, 1.26]; 5 target dots: ES = 0.90, 95% CI [0.20, 1.62). In summary, these results show that for a tracking duration of 5 s, at small target-dot numbers (i.e., 3 and 4) patients with mild TBI performed as well as control participants, but on average performed worst when they were required to track more dots.

For a tracking duration of 10 s, a two-way ANOVA again showed a main effect of both group (F (1, 98) = 22.60, p < 0.0001, η^2^_p_ = 0.19) and number of target dots (F (2, 98) = 42.37, p < 0.0001, η^2^_p_ = 0.46) and no significant interaction effect (F (2, 98) = 0.18, p = 0.83, η^2^_p_ = 0.004). Here, post hoc comparison’s tests showed that patients with TBI performed worse than control participants for all target numbers (3 target dots: MD = 1.59, p = 0.004; 4 target dots: MD = 1.24, p = 0.03; 5 target dots: MD = 1.21, p = 0.04). Effect size calculations showed large group differences for all target dot-numbers (3 target dots: ES = 0.97, 95% CI [0.27, 1.68]; 4 target dots: ES = 0.85, 95% CI [0.15, 1.55]; 5 target dots: ES = 1.25, 95% CI [0.52, 1.98]).

**Figure 4:**
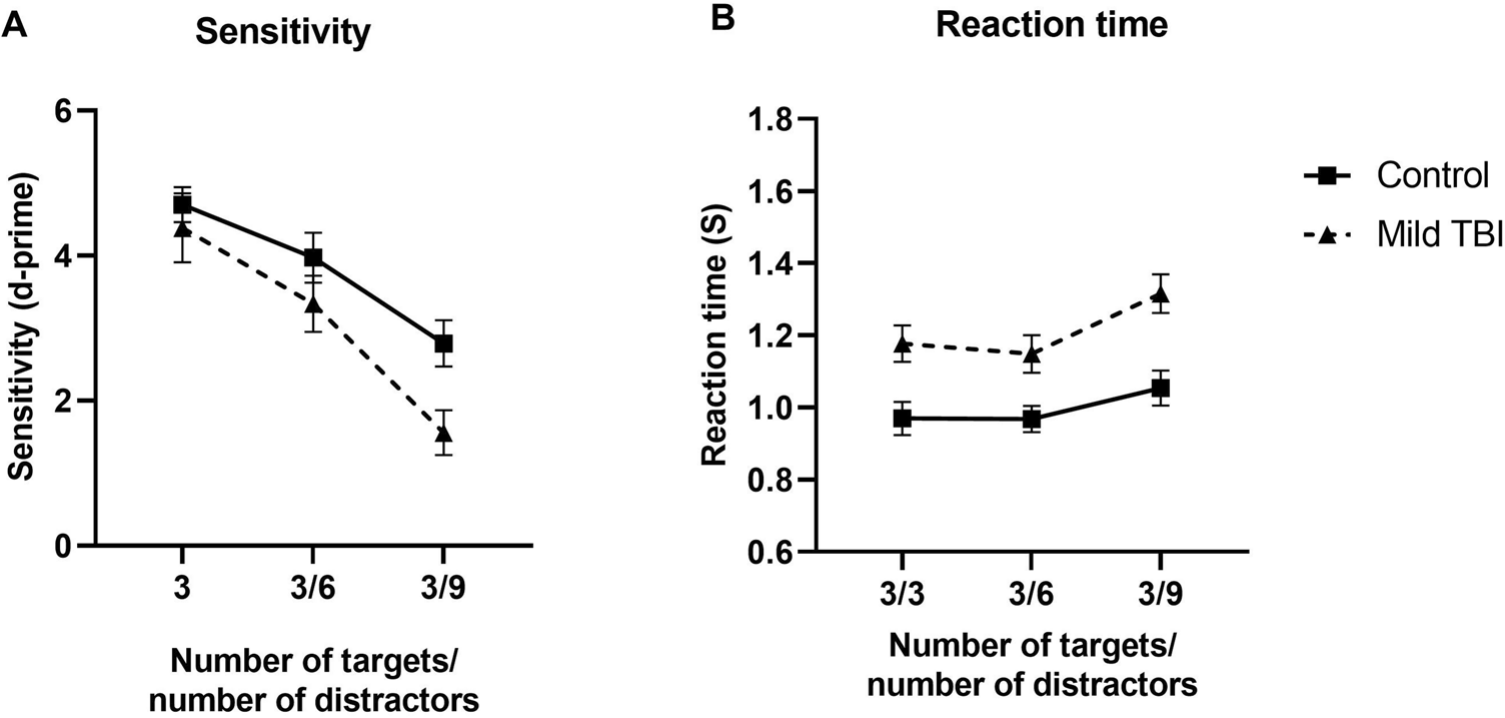
Sensitivity (**A**) and reaction time (**B**) as a function of the number of distractor dots for control participants and patients with mild TBI (different symbols). Error bars indicate 1 standard error of the mean.

In Fig. 4A, sensitivity data (average d-prime) for patients with mild TBI and control participants are plotted for conditions in which the number of distractor dots was varied. Overall, the sensitivity decreased with increasing number of distractors and patients with mild TBI performed poorer on average. Indeed, a 2-way ANOVA analysis revealed a main effect of group (F (1, 97) = 6.66, p = 0.01, η^2^_p_ = 0.07) and target dot number (F (2, 97) = 24.00, p < 0.0001, η^2^_p_ = 0.33). There was no significant interaction (F (2, 97) = 0.89, p = 0.41, η^2^_p_ = 0.01). Again, Sidak, post-hoc analysis comparing performance between the two groups, confirmed that group differences between patients with mild TBI and control participants are most evident when the number of distractor dots were large (3 distractor dots: MD = 0.32, p = 0.88; 6 distractor dots: MD = 0.64, p = 0.48; 9 distractor dots: MD = 0.23, p = 0.04). Confirming this trend, effect sizes systematically increased with the number of distractor dots (3 distractor dots: ES = 0.22, 95% CI [− 0.45, 0.89]; 6 distractor dots: ES = 0.44, 95% CI [− 0.24, 1.12]; 9 distractor dots: ES = 0.97, 95% CI [0.26, 1.68]).

#### Reaction time

Average reaction time (for trials in which participants performed a correct judgement, i.e., correctly judged that the cue dot was or was not a target) data for patients with mild TBI and control participants are shown in Fig. 3C and D. As can be seen in these figures, overall, reaction times increase with the number of target dots; however, patients with mild TBI consistently required longer to make a judgement across all conditions relative to control participants. A similar analysis was conducted as in sensitivity. For tracking duration of 5 s, a 2-way ANOVA analysis yielded a significant main effect of group (F (1, 97) = 27.32, p < 0.0001, η^2^_p_ = 0.22) and significant main effect of target dot number (F (2, 97) = 7.035, p = 0.001, η^2^_p_ = 0.13). There was no significant interaction (F (2, 97) = 0.0096, p = 0.99, η^2^_p_ = 0.0002). Sidak’s multiple comparisons test shows that patients with mild TBI took significantly longer than control participants to correctly discriminate the target dots for all to be tracked target dot numbers (3 target dots: MD = 0.21, p = 0.012; 4 target dots: MD = 0.23, p = 0.007; 5 target dots: MD = 0.23, p = 0.01). Effect size calculations revealed that these differences were large for all number of target dots (3 target dots: ES = 1.17, 95% CI [0.45, 1.89]; 4 target dots: ES = 1.17, 95% CI [0.45, 1.89]; 5 target dots: ES = 0.94, 95% CI [0.24, 1.65]). For a tracking duration of 10 s, a 2-way ANOVA analysis yielded a significant main effect of group (F (1, 98) = 28.40, p < 0.0001, η^2^p = 0.23) and a significant main effect target dot number (F (2, 98) = 3.96, p = 0.02, η^2^p = 0.08). There was no significant interaction effect (F (2, 98) = 0.057, p = 0.94, η^2^_p_ = 0.001). Sidak’s multiple comparisons test shows that patients with mild TBI responded significantly longer than control participants and Cohen’s d effect size calculations indicated that these differences were all large regardless of the number of target dots (3 target dots: MD = 0.29, p = 0.004, ES = 1.32, 95% CI [0.58, 2.06]; 4 target dots: MD = 0.28, p = 0.006, ES = 1.04, 95% CI [0.32, 1.75]; 5 target dots: MD = 0.25, p = 0.01, ES = 0.95, 95% CI [0.25, 1.66]).

In Fig. 4B, average reaction time data for patients with mild TBI and control participants are plotted for conditions in which the number of distractor dots was varied. Two-way ANOVA revealed a main effect of group (F (1, 96) = 30.36, p < 0.0001, η^2^_p_ = 0.24) and distractor element number (F (2, 96) = 4.170, p = 0.02, η^2^_p_ = 0.08). There was no significant interaction (F (2, 96) = 0.3675, p = 0.69, η^2^_p_ = 0.008). Sidak’s multiple comparisons test shows that patients with mild TBI significantly slower than control participants for all different distractor dot number (3 distractor dots: MD = 0.21, p = 0.008; 6 distractor dots: MD = 0.18, p = 0.03; 9 distractor dots: MD = 26, p = 0.0005). Again, effect sizes indicated that there was a large difference between the performance of patients with mild TBI and control participants for all distractor dot number conditions (3 distractor dots: ES = 1.17, 95% CI [0.45, 1.89]; 6 distractor dots: ES = 1.10, 95% CI [0.38, 1.81]; 9 distractor dots: ES = 1.27, 95% CI [0.54, 2.01]).

### Correlations between the performance on the visual attention task symptoms scores in patients with mild TBI

**Figure 5:**
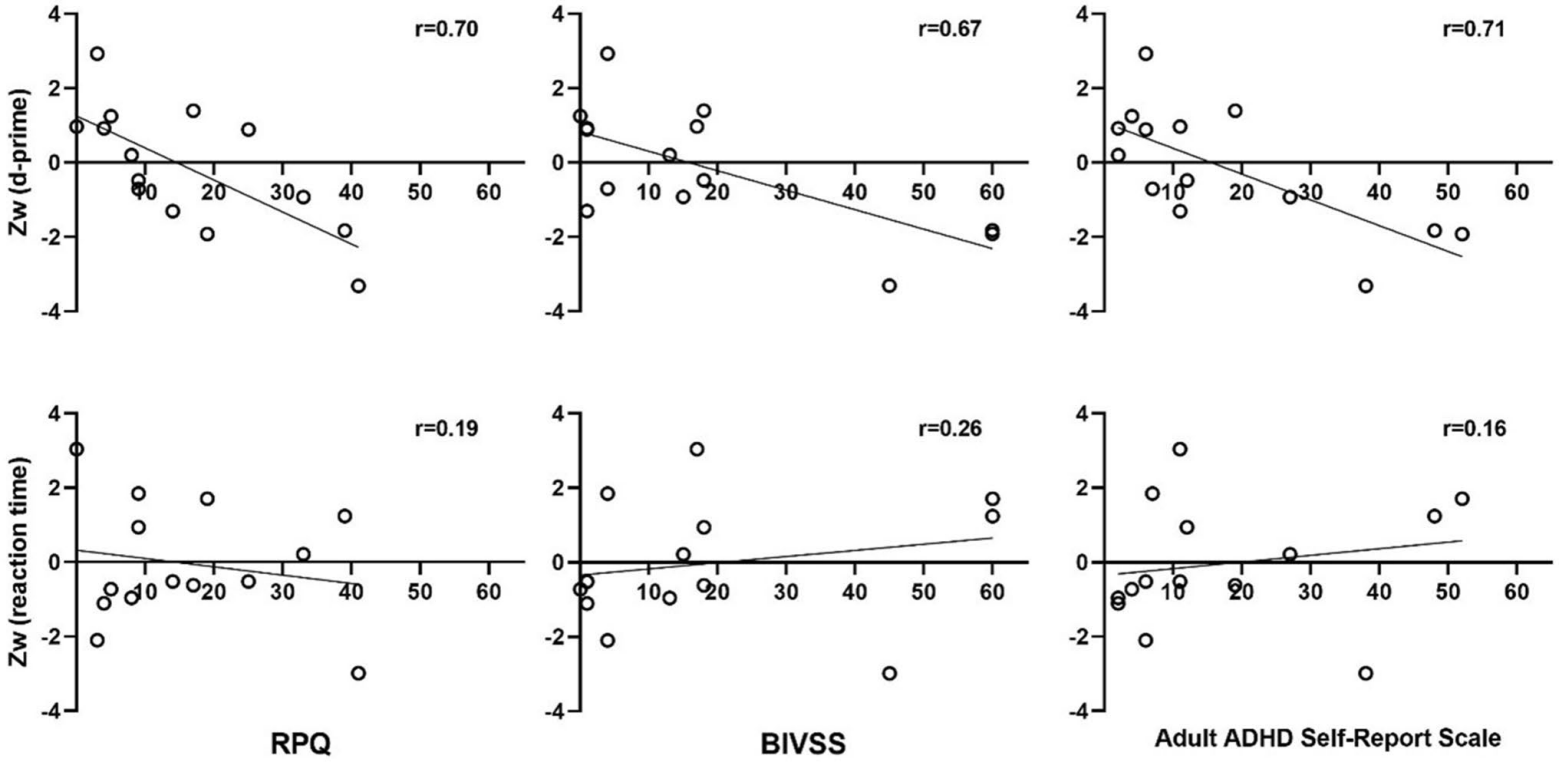
The association between the overall MOT performance (sensitivity and reaction time) as measured by weight-averaged Z-scores and symptoms scores for RPQ, BIVSS questionnaire, and the Adult ADHD Self-report Scale. Circle markers in each graph indicate individual subjects and the solid line represents the line of best fit.

We determined whether there is an association in TBI symptoms as measured by the RPQ, BIVSS and Adult ADHD Self-report Scale with performance on our MOT task among patients with mild TBI. As shown in Fig. 5, overall sensitivity in performing MOT task (represented by the combined Z-score of the 3 MOT conditions) was significantly correlated with all three measures of TBI symptoms (RPQ: r = − 0.70, p = 0.005; BIVSS: r = − 0.67, p = 0.009; Adult ADHD Self-report Scale: r = − 0.71, p = 0.004). In particular, higher TBI symptoms, as reported by the three questionnaires, were associated with poorer sensitivity score. In contrast, there was no significant correlation between the overall performance score for reaction time and all three measures of TBI symptoms (RPQ: r = − 0.16, p = 0.53; BIVSS: r = 0.22, p = 0.46; Adult ADHD Self-report Scale: r = 17, p = 0.53).

### IQ and MOT performance

Since tracking multiple moving objects has been reported to be different among individuals, a linear regression analysis was conducted to determine whether IQ (measured using WASI-II) influences the performance on MOT task. For control participants, there was no significant association/change in the overall performance score for sensitivity (represented by the combined Z-score of the 3 MOT conditions) and IQ score (F (1, 18) = 1.50, p = 0.24). Similarly, for patients with mild TBI, there was no significant change in the overall performance score for sensitivity and IQ score (F (1, 13) = 0.85, p = 0.37). Together, performance on the MOT task did not influence the performance in both groups in the current study.

### Effect of post-injury period

**Figure 6:**
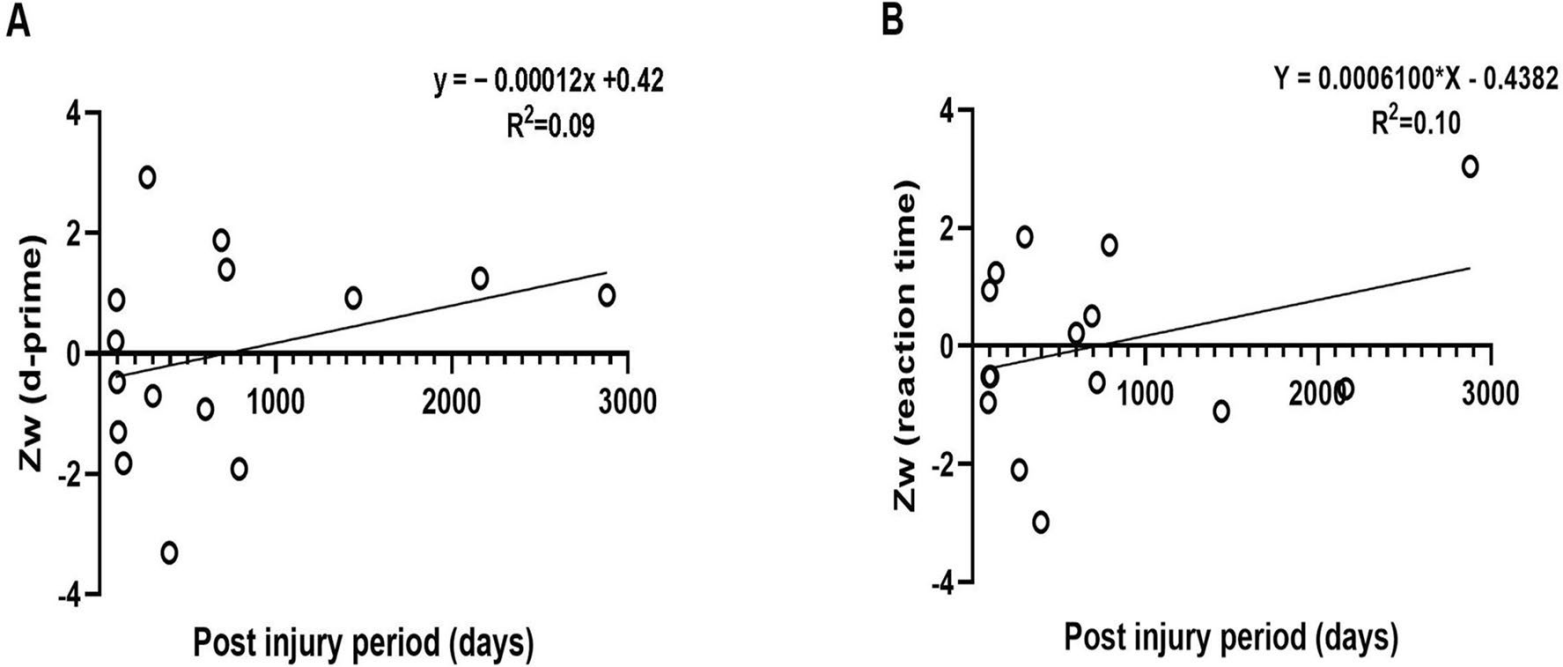
A linear regression analysis between the overall MOT performance (sensitivity and reaction time) as measured by weight-averaged Z-scores and post-injury periods. The solid line represents the line of best fit.

A linear regression analysis was conducted to determine whether post-injury period influences the performance on MOT task. The results of the regression analyses are presented in Fig. 6. There was no significant change in the overall performance score for sensitivity and the post-injury period (F (1, 13) = 1.36, p = 0.27) as shown in Fig. 6A. Similarly, there was no significant change in the overall performance score for reaction time and the post-injury period (F (1, 13) = 1.48, p = 0.25) as shown in Fig. 6B. Together, performance on the MOT task as measured by sensitivity (d-prime) and reaction time did not improve as a function of years following the initial injury. Note that, it cannot be entirely guaranteed that the symptoms for TBI patients with long post injury periods are from the original or a subsequent injury. We interviewed all TBI patients in the present study to confirm that they did not acquire a subsequent brain injury. Importantly, though excluding TBI patients with post injury periods greater than 1000 days did not change the outcomes of this analysis and in the interest of reporting all data they were left in the analysis.

## Discussion

The primary aim of the study was to assess the ability of patients with mild TBI to maintain visual attention using a MOT task, and whether their performance was significantly altered compared to normal participants without TBI. In separate conditions, the number of target dots, distractor dots and tracking duration were treated as independent variables to investigate how dividing attention to selectively track multiple objects is susceptible to distraction and duration in patients with TBI. In addition, we investigated whether the ability to track multiple moving objects is associated with mild TBI symptoms as measured using three standardized questionnaires. We find that patients with mild TBI can track multiple objects, but overall performance (as indicated by sensitivity and reaction time outcome measures) was poorer compared to control participants, particularly for conditions in which the tracking duration was long and the number of target and distractor dots were large (see Figs. 3 and 4).

We find that MOT performance (sensitivity and reaction times) is affected by the duration of the tracking period. When the tracking duration was 5 s, patients with mild TBI only performed poorer than control participants when there was a large number of target dots were required to be tracked. These findings perhaps suggest that the maximum attentional capacity is affected by TBI. Whilst patients with mild TBI can divide their attention to track multiple objects (agreeing with the findings of Schneider and Gouvier^36^), their performance is not equal to control participants (i.e., they have poorer sensitivity and slower reaction times) and is susceptible to decay when tracking a large number of elements for short durations. However, for longer tracking durations (10 s) poorer mild TBI group performance was observed regardless of the number of target dots that needed to be tracked. Importantly, these findings suggest that patients with mild TBI might have problems with the ability to maintain continuous visual attention for long periods of time.

Visual working memory has been shown to be important to multiple objects tracking, as demonstrated by Oksama and Hyönä^40^ and has been found to be impaired following TBI^53–55^. It is possible that deficits in visual working memory can explain the findings of the present study. However, it is important to note that both groups performed similarly for short tracking duration, but poorer for long tracking duration, and this suggests a deficit in sustaining visual attention. Further studies are needed to confirm whether poorer MOT performance following TBI is due to a deficit in visual working memory and or sustaining visual attention.

The ability to track multiple moving objects has been reported to be different among individuals^40,56–58^ and this is reported to be positively correlated to individual differences such as IQ^40,59^. In the current study, we did not observe a significant correlation between IQ scores and the overall performance in the MOT task (see results). This minimizes the likelihood that IQ might be a confounding factor in MOT performance among the study participants. Where perhaps IQ might impact our results is if there were a difference in IQ between control participants and TBI patients. However, as reported in Table 1, the IQ of both groups was similar and not significantly different. Accordingly, it is unlikely that IQ is a major contributing factor in accounting for a difference in MOT performance between both groups.

We find that whilst the performance (task sensitivity and reaction times) of both patients with mild TBI and control participants decreased with the number of distractor dots, the magnitude of change was greater for TBI patients. This perhaps suggests that TBI affects selective visual attention and reflects the degree to which they can attend to target dots whilst ignoring distractors. Poorer performance at high distractor dots number suggests impairments in the ability to selectively attend to target objects. Our findings are supported by numerous studies that have reported deficits in selective attention following TBI and the detrimental effect of distractors on selective attention^23–25,60^. For example, using a visual search task^25^, showed that patients with mild TBI were more affected by increases in the number of distractors relative to control participants. Our findings confirm that this deficit is seen with moving objects, and when maintained attention unlike visual search paradigms which assess the initial allocation of attention.

Various models of MOT indicate that an observer can successfully attend multiple moving objects for a certain period of time by either perceptually groups object into a single global object, rapidly switching attention between the locations of the objects, or splitting attention between the different target locations^40,42,46,47^. Our finding showed that the ability to maintain attention to MOT is distrusted for specific task conditions, including increased number of targets, tracking duration and number of distractors. We cannot conclude whether this is a deficit in local or global tracking strategies and recommend that future studies might incorporate eye tracking to assess eye movement patterns during MOT to investigate whether observers alternate attention on local elements or use a global approach such as tracking objects based on their average or centroid location.

The present study also examined the relationship between performance on the MOT task and symptoms scores in patients with mild TBI. This correlational analysis was not conducted for control participants since there was no variability in symptoms scores. Significant and large correlations were found between overall sensitivity on the MOT task and the three different questionnaires. These questionaries have been designed and validated to assess the concussion, visual, and attention related symptoms, and we find that individuals with high symptom scores (i.e., report more deficits in attention) also had lower sensitivity in the MOT task. This finding confirms that visual attention deficit is a major deficit following TBI and can have an impact on function particularly and as confirmed by the present study on motion tracking.

In contrast, we observe that the overall reaction time did not correlate with any symptoms score for all the three different questionaries. We suggest that having more symptoms may be associated with poorer sensitivity, but not necessarily slower performance on the MOT task. Our result is consistent with the study by Ziino and Ponsford^61^ who reported that the Visual Analogue Scale for Fatigue, which measures subjective fatigue level, correlated significantly with performance accuracy on a selective attention task, but not with reaction time.

The association between symptoms reported by patients with TBI and neurobehavioral performance as measured by symptoms questionnaires has been investigated previously, but the strength of the relationship can be variable. This might be because neurobehavioral tests typically consist of multiple domains, such as memory, attention, and executive functioning, with different scoring procedures, such domains are not directly related to the behavioural measure. Moreover, some studies assess performance with different sets of symptoms, such as memory, anxiety, and depression related symptoms^62–64^. For example, Arcia and Gualtieri^63^ showed that performance on neurobehavioral tests was only associated with memory difficulties and similarly^64^ reported no significant association between performance and symptoms among patients with mild TBI. We find a similar result with our correlation analysis between the outcome of the symptoms questionnaire and reaction time. Note that the lack of association may be accounted for by the fact that the questionnaires used in the present study specifically measure attention related symptoms and not necessarily the speed of attentional processing, which reaction time is indicative of.

Patients with mild TBI not only exhibit a variety of symptoms but also have continuing deficits in visual attention even years after the initial injury. This was evident from the regression analyses (see Fig. 6), which did not find an association between the overall performance in the MOT task and post-injury period. Our findings contradict with known recovery period of cognitive deficits following mild TBI which is less than 3 months^65,66^. However, it is known that not all patients with mild TBI recover with in the first 3 months, but may take up to 3 years^67,68^ and some functions may recover faster than others. Our findings suggest that deficits in visual attention may be enduring and last for more than 1 year in some individuals. Though future studies are needed to understand whether and how this process recovers.

Our findings provide evidence of impairment in the ability to maintain visual attention in tasks that required attentive tracking, such as driving and scanning the environment. This impairment might be a factor in the increased risk of accidents among drivers in patients with mild TBI as the ability to maintain visual attention plays an important role in driving and is frequently required to continually track moving objects in the visual scene^69,70^. Additionally, our results show that patients with mild TBI do not only have impaired ability to maintain visual attention but also demonstrated increased difficulty when attending more visual objects and when there is a large number of distractors.

## Limitations

Although our results showed the ability to maintain visual attention may be altered by mild TBI, a number of methodological limitations should be considered. Though the present study establishes a deficit in MOT following TBI, a limitation of this study is related to a small number of participants, limits the generalizability of this study. The inclusion of more participants in the future, and different TBI severities, will be informative in understanding the scope and degree to which the ability to maintain visual attention is affected by brain injury. Moreover, a longitudinal design may by informative in determining whether the visual attention deficits reported in our study resolve or continue to persist over time. Additionally, note that the present study was case–control by design, as we were interested in establishing whether TBI leads to deficits in MOT. However, future studies may wish to employ participants with different deficits, such as those due to other non-brain injuries (e.g., orthopedic injury) to establish whether MOT deficits might be due to other shared conditions/symptoms such as chronic fatigue and pain.

Mild TBI populations can be heterogenous in nature in terms of aetiology, post-injury period, and symptomatology. In our sample, the majority of mild TBI was due to motor vehicle accidents, however the mechanism of injury can include direct impact, rapid acceleration–deceleration and blast force. The cause of TBI might result in different TBI related pathologies and different degrees of functional deficits (including MOT performance), which could not be confirmed in the present study as the majority did not have CT or MRI data on which to assess the actual impact on brain physiology and function. Again, future studies may wish to establish whether MOT performance is differently affected by the type of brain injury and compare with available CT or MRI data.

Additionally, we did not recruit individuals with acute mild TBI (less than 3 months) since they were instructed to avoid high visual or physical tasks. It might be expected that in this group, deficits in MOT to be more impaired, but this assertion requires future investigation, as is the possibility of greater deficits for individual with greater TBI severities.

Finally, our study approach is ‘case–control’ in design and similar to many studies that have sought to understand deficits in visual cognition following TBI by comparing performance with a group of appropriately matched non-TBI participants as controls. The utility of such an approach is largely limited to highlighting the potential impact of TBI on brain function, but within-subject designs are needed to establish a more definitive causal relationship in which comparisons in performance is made in individuals pre and post TBI. However, such designs are not always feasible to conduct in human populations (as opposed to animal studies in which controlled TBI can be performed) as it is not possible to anticipate or predict who and when an individual will acquire a TBI to a level of certainty in which pretesting can be effectively done. Such an approach can be only achieved if mass pre-testing were to be undertaken on the expectation that a small percentage of those tested acquire a TBI. Given that the global annual rate of acquiring TBI is estimated to be approximately 1–1.5% of population^3^, substantial pre-testing involving thousands of ‘normal’ participants may be necessary to obtain subsequent sufficient numbers of TBI for a pre-post comparison. This is obviously entirely beyond the scope of the present study, which was exploratory and sought to establish whether there is a deficit in MOT in a mild TBI group.

## Conclusion

The current study provided evidence that shows that mild TBI affects the ability to maintain visual attention. In general, patients with mild TBI performed poorer (in terms of sensitivity and reaction) in tracking multiple objects as compared to control participants and are more susceptible to distraction and longer periods of concentration. These findings perhaps might account for or associated with symptoms of visual attention deficits and, more importantly, behavioral deficits in tasks that require maintaining attention on multiple objects over extended durations.

## Method

The study procedures were approved and performed in compliance with the Human Research Ethics Committee of the University of New South Wales, Sydney (UNSW HREC: #HR200527) and the Regional Research Ethics Committee Qassim Region, Buraydah (#1442-874691). All participants were informed of the study aim and gave their written informed consent.

### Participants

Our systematic review and meta-analysis^52^ indicated that the effect size for visual attention deficits following TBI is large and estimated to be 0.92. The sample size assuming a power of 0.8 is estimated to be 32 participants equally divided into two groups (16 TBI and 16 healthy control participants). However, in conducting the study, a small number of TBI patients withdrew during the and in total fifteen adults with mild TBI (31.1 ± 4.9 years, 11 males, 23.9 ± 26.7 months post-injury) and 20 control participants (28.9 ± 4.2 years, 13 males) participated in the present study. Patients with mild TBI were recruited through various Optometry clinics and Quraif rehabilitation centre in Qassim region of Saudi Arabia. Information indicating injury severity was obtained from medical records and included the Glasgow Coma Scale (GCS), Duration of Loss of Consciousness (LOC), and Duration of Post-Traumatic Amnesia (PTA). Not all severity measures were available for all participants. Other variables, such as post-injury period and injury mechanism, were also collected from the participants.

Patients with mild TBI were first interviewed to determine their ability to perform the visual attention task in which the participant must be able to score at least 75% in the practice trials. Participants were excluded if they were not able to perform the practice trials, had a binocular visual acuity worse than 6/12, or neurodegenerative diseases such as Parkinson’s disease and Alzheimer’s disease. Participants were also excluded if they report any neurodevelopmental disorders, including autism spectrum disorders (ASD) or attention deficit hyperactivity disorder (ADHD). Age matched control participants were recruited through advertising (distribution of flyers) in the same Qassim region. Control participants were excluded if they reported a history of head trauma or any neurological disorders. All participants were not native English speakers but learnt English before the age of 12.

### Symptoms

Participant symptoms were assessed using 3 different standardized questionnaires that measure general, visual, and attention related symptoms. All questionnaires were self-administered, and completed on the same day on which they participated in the study. First, The Rivermead Post-Concussion Symptoms Questionnaire (RPQ) was used to assess general symptoms that persist after head injury, including cognitive, emotional, and physical symptoms on a 16-item scaled questions^71^. Participants were asked to score their symptoms on a 5-point scale ranging from 0 (not experienced at all) to 4 (a severe problem). Secondly, the Brain Injury Vision Symptom survey (BIVSS) questionnaire was used to vision symptoms related to TBI on a 28-item scaled questions^72^. Participants were asked to score their symptoms on a 5-point scale ranging from 0 (never) to 4 (always). Finally, the Adult ADHD Self-report Scale was used to detect Attention deficit hyperactivity disorder ADHD in the general population^73^. This questionnaire provided another indication of any potential deficits in attention following TBI and akin to adult ADHD. The Adult ADHD Self-report Scale comprises18 items and the participant were asked to score their symptoms on a 5-point scale ranging from 0 (never) to 4 (very often).

### Intelligence

The Wechsler Intelligence Scale WASI-II was used to assess the participant’s general intelligence (IQ) across different aspects of intelligence^74^. The WASI-II comprised of 4 subtests, including Block Design (BD), Vocabulary (VC), Matrix Reasoning (MR), and Similarities (SI). Performance on all 4 subtests was scaled into the conventional IQ unit as per the instructions and conventions of the test. We measured IQ to rule the possibility that differences in task performance may be due to IQ; that is the general aptitude to follow instructions and correctly perform tasks.

### Vision tests

We also assessed the vision of all participants via standard optometric tests for visual acuity and visual field. Monocular and binocular distance visual acuity was measured with the participant’s distance habitual correction using the Freiburg Vision Test (‘FrACT’), at a distance of 3 m^75^. This test of visual acuity involved the presentation of a Landolt-C on a monitor at one of 4 orientations. The participants were asked to respond to the orientation of the Landolt-Cs using a keypad. The size of the optotype was systematically decreased following a modified PEST staircase procedure to determine the acuity threshold. Near binocular visual acuity was measured with the participant’s distance habitual correction using “The Eye Handbook” mobile application presented on an iPad. The near vision chart was viewed from 40 cm. The participant was instructed to read from the largest row letter by letter and was asked to guess the letters when they were not sure. Visual acuity was recorded using the by-letter scoring system, where each letter was equal to 0.02 logMAR.

Participants also completed confrontation visual fields test; whereby static, single-quadrant counting was used to identify any gross visual field defect in the peripheral visual field. In addition, multiple vision screening tests were performed to rule out vision related disorders, including pupil reaction, unilateral and alternating cover test, and ocular motility^76–78^.

### Multiple object tracking task (MOT)

#### Stimulus

Stimuli consisted of a number of identical white dots (Weber Contrast = 0.8) subtending a visual angle of 0.57 degree, presented on a grey rectangular (17.1 × 12.1°) grey background (14 cd/m^2^) at a viewing distance of 70 cm (see Fig. 1). The stimulus was generated using MATLAB 2018 software^79^, with the Psychophysics Toolbox extensions^80^. The screen resolution was 1024 × 768 pixels on a 27-inch screen with a refresh rate of 60 Hz, at the viewing distance of 70 cm.

The initial position of each dot, as well as the directions, was generated randomly. At the beginning of each trial, a number of dots were randomly selected as target dots and their location briefly cued by changing their colour to black (4 cd/m^2^) for 2 s. After this period, they reverted to their original colour (white) and were indistinguishable from distractor dots, and all stimuli underwent random motion at a speed of 2.7°/s for a fixed tracking duration. Stimuli were presented from overlapping and a circular buffer (radius of 0.5°) was applied to all stimuli to prevent their edges from merging with each other.

#### Procedure

Participants used their habitual vision correction during the task and were seated 70 cm from a display monitor. Before starting the MOT task, each participant was given a verbal explanation of the task sequence followed by 10 practice trials to familiarize themselves with the task. Participants performed the experiment using a chinrest to stabilize head movements and maintain a constant viewing distance from the monitor.

In the main experiment, the ability to track multiple objects was assessed in conditions in which the number of target elements or distractors was systematically changed. In Condition 1, the number of target elements were systematically changed in different trials and participants were required to track 3, 4 and 5 target dots with an equal number of distractor dots. After a tracking duration of either 5 or 10 s, all dots stopped moving, and the stimulus was replaced by a static white noise mask presented for 100 ms to minimize any after-effects, and to signal the end of the tracking period. After which, a randomly chosen dot was cued (by changing its colour to black), and the task of the participant was to indicate (by pressing keys on a keyboard) whether it was a target or distractor dot. Note that across all trials, there was equal probability that the cued dot was a target or distractor. There were 6 conditions in total, in which the 3 target levels were repeated twice for tracking durations of 5 and 10 s.

In Condition 2, the number of target dots was fixed at 3 and the number of distractor dots was systematically changed from 3, 6 and 9 dots. Only a tracking duration of 5 s was used in this condition. There were 8 experimental sub conditions in total and these conditions consisted of 160 trials (20 trials per condition) presented in a randomized order and took approximately 40 min to complete. To minimize fatigue from repetition, brief breaks were offered in between conditions, and the start of the next condition was prompted by the participant.

#### Outcome measures

There were two outcome measures: sensitivity (d-prime), and reaction time RT (in seconds). These two outcome measures are typically used as performance measures of visual attention. The sensitivity in discriminating whether the cued dot at the end of a trial was a signal dot was quantified as Signal Detection Theory Sensitivity Index (d′) and was computed from the hit and false alarm rates based on the following equation:

$${d}^{{\prime}}=z\left(H\right)-z\left(FA\right).$$

where z is the z-transforms, H is the hit rate, and FA is the false alarm, using MATLAB functions that were part of the Palamedes toolbox^81^. Occasionally, perfect performance was obtained by control participants, and here we applied a correction factor as per the methods and justification of Brown and White in which the proportion of hit and false alarms were set at 0.99 and 0.01 respectively^82^. Reaction time for each trial was recorded, and represented the time taken for the participant to make a response (by pressing the keyboard) after an element was cued. Reaction time is commonly used as a measure of information processing speed, which reflects the speed of different cognitive processes, including attention and decision making. As noted above, deficits in processing speed, as measured by reaction time, have been shown to be affected by TBI^16^. On other hand, task sensitivity measures how successfully the participant is able to perform the task in distinguishing between target and distractors.

### Statistical analysis

Statistical analyses were conducted using GraphPad prism (Graph Pad Software Inc., San Diego, CA). D’Agostino and Pearson’s test confirmed the data for different conditions were normally distributed. Three analyses were conducted. Firstly, 2-way ANOVA with factors of tracking load (3, 4, and 5) and group (mild TBI vs. control) for the tracking duration of 5 and10 seconds were used to compare the different tracking loads between mild TBI and control groups. This analysis was conducted for both outcome measures. Secondly, 2-way ANOVA with factors number of distractor dots (3,6, and 9) and group (mild TBI vs. control) were used to compare the number of distractor dots between mild TBI and control groups. This analysis was also conducted for sensitivity and reaction time data. We also calculated Cohen’s d effect sizes for each MOT condition by dividing the difference between the mean of the performance on the MOT task of patients and controls by the pooled standard deviation (SD)^83^. Thirdly, independent samples t-tests (parametric) or Mann–Whitney tests (non-parametric) were used to compare age, symptoms scores, and IQ between mild TBI and the control group.

We also assessed whether there was a significant relationship between MOT tracking performance and scores from our 3 symptoms questionnaires (i.e., RPQ, BIVSS, and Adult ADHD Self-report Scale). Since there were different conditions in the MOT task, an overall performance index for the sensitivity and reaction time for each patient with mild TBI was calculated by converting their individual results for the 3 different MOT conditions into Z-scores (using the mean and standard deviation values for the normal control group), and then combining Z scores using a weighted sum following the formula:

$${Z}_{W= }\frac{\frac{{W}_{A}}{{W}_{A}+{W}_{B}+{W}_{C}}\times {Z}_{A}+\frac{{W}_{B}}{{W}_{A}+{W}_{B}+{W}_{C}}\times {Z}_{B}+\frac{{W}_{C}}{{W}_{A}+{W}_{B}+{W}_{C}}\times {Z}_{C}}{\sqrt{{\left(\frac{{W}_{A}}{{W}_{A}+{W}_{B}+{W}_{C}}\right)}^{2}}+{\left(\frac{{W}_{B}}{{W}_{A}+{W}_{B}+{W}_{C}}\right)}^{2}+{\left(\frac{{W}_{C}}{{W}_{A}+{W}_{B}+{W}_{C}}\right)}^{2}}$$

where Z_A_, Z_B_ and Z_C_ represent the standardised performance on the different MOT tasks, and W_A,_ W_B,_ and W_C_ refer to the weights which equalled to 1. The combined Z score (Z_w_) for each participant was then correlated (Pearson R) with their outcomes for the three different symptom scores.

## References

[1] Statements Q (2009). VA/DoD clinical practice guideline for management of concussion/mild traumatic brain injury. J. Rehabil. Res. Dev..

[2] Menon DK (2010). Position statement: Definition of traumatic brain injury. Arch. Phys. Med. Rehabil..

[3] Peterson, A.B. *et al*. *Surveillance Report of Traumatic Brain Injury-Related Emergency Department Visits, Hospitalizations, and Deaths, United States, 2014*. (2019). 10.15585/mmwr.ss6609a1 PMC5829835 28301451

[4] Teasdale G, Jennett B (1974). Assessment of coma and impaired consciousness: A practical scale. The Lancet.

[5] Blennow K, Hardy J, Zetterberg H (2012). The neuropathology and neurobiology of traumatic brain injury. Neuron.

[6] McCrory P (2005). Summary and agreement statement of the 2nd International Conference on Concussion in Sport, Prague 2004. Br. J. Sports Med..

[7] Alexander, M. P. Mild traumatic brain injury: pathophysiology, natural history, and clinical management. *Neurology*. (1995). 10.1212/wnl.45.7.1253 7617178

[8] Smith ST (2017). Postconcussion syndrome: An overview for clinicians. Psychiatr. Ann. J..

[9] Marshall S (2012). Clinical practice guidelines for mild traumatic brain injury and persistent symptoms. Can. Fam. Physician.

[10] Ciuffreda KJ (2007). Occurrence of oculomotor dysfunctions in acquired brain injury: A retrospective analysis. Optom. J. Am. Optom. Assoc..

[11] Kapoor N, Ciuffreda KJ (2002). Vision disturbances following traumatic brain injury. Curr. Treat. Options. Neurol..

[12] Barnett BP, Singman EL (2015). Vision concerns after mild traumatic brain injury. Curr. Treatm. Opt. Neurol..

[13] Ryan LM, Warden DL (2003). Post concussion syndrome. Int. Rev. Psychiatry.

[14] McCrea M (2009). An integrated review of recovery after mild traumatic brain injury (MTBI): Implications for clinical management. Clin. Neuropsychol..

[15] Greenwald BD, Kapoor N, Singh ADJ (2012). Visual impairments in the first year after traumatic. Brain Inj..

[16] Mathias JL, Wheaton P (2007). Changes in attention and information-processing speed following severe traumatic brain injury. Neuropsychology.

[17] Rabinowitz AR, Levin HS (2014). Cognitive sequelae of traumatic brain injury. Psychiatr. Clin. N. Am..

[18] Yantis S, Johnston JC (1990). On the locus of visual selection: Evidence from focused attention tasks. J. Exp. Psychol..

[19] Schneider W, Shiffrin RM (1977). Controlled and automatic human information processing: I. Detection, search, and attention. Psychol. Rev..

[20] Robertson IH (1996). The structure of normal human attention: The test of everyday attention. J. Int. Neuropsychol. Soc..

[21] Posner MI, Petersen SE (1990). The attention system of the human brain. Annu. Rev. Neurosci..

[22] Fisk GD (2002). Useful field of view after traumatic brain injury. J. Head Trauma Rehabil..

[23] Geldmacher DS, Hills EC (1997). Effect of stimulus number, target-to-distractor ratio, and motor speed on visual spatial search quality following traumatic brain injury. Brain Inj..

[24] Hills EC, Geldmacher DS (1998). The effect of character and array type on visual spatial search quality following traumatic brain injury. Brain Inj..

[25] Schmitter-Edgecombe M, Kibby M, Michelle K (1998). Visual selective attention after severe closed head injury. J. Int. Neuropsychol. Soc..

[26] Bate AJ, Mathias JL, Crawford JR (2001). The covert orienting of visual attention following severe traumatic brain injury. J. Clin. Exp. Neuropsychol..

[27] Cremona-Meteyard S (1992). Covert orientation of visual attention after closed head injury. Neuropsychologia.

[28] Pavlovskaya M (2007). Hemispheric visual atentional imbalance in patients with traumatic brain injury. Brain Cogn..

[29] van Donkelaar P (2005). Attentional deficits in concussion. Brain Inj..

[30] Wu Y (2020). Multiple component analysis of attention early after complicated mild traumatic brain injury: A prospective cohort study. J. Rehabil. Med..

[31] Malojcic B (2008). Consequences of mild traumatic brain injury on information processing assessed with attention and short-term memory tasks. J. Neurotrauma.

[32] Slovarp L, Azuma T, LaPointe L (2012). The effect of traumatic brain injury on sustained attention and working memory. Brain Inj..

[33] McIntire A (2006). The influence of mild traumatic brain injury on the temporal distribution of attention. Exp. Brain Res..

[34] Robertson K, Schmitter-Edgecombe M (2017). Focused and divided attention abilities in the acute phase of recovery from moderate to severe traumatic brain injury. Brain Inj..

[35] Schneider JJ, Gouvier WD (2005). Utility of the UFOV test with mild traumatic brain injury. Appl. Neuropsychol..

[36] Walz JA (2021). Visuospatial attention allocation as an indicator of cognitive deficit in traumatic brain injury: A systematic review and meta-analysis. Front. Hum. Neurosci..

[37] Madden DJ (1992). Selective attention and visual search: Revision of an allocation model and application to age differences. J. Exp. Psychol. Hum. Percept. Perform..

[38] Müller HJ, Krummenacher J (2006). Visual search and selective attention. Vis. Cogn..

[39] Pylyshyn ZW, Storm RW (1988). Tracking multiple independent targets: Evidence for a parallel tracking mechanism. Spat. Vis..

[40] Oksama L, Hyönä J (2004). Is multiple object tracking carried out automatically by an early vision mechanism independent of higher-order cognition? An individual difference approach. Vis. Cogn..

[41] Feria CS (2012). The effects of distractors in multiple object tracking are modulated by the similarity of distractor and target features. Perception.

[42] Kahneman D, Treisman A, Gibbs BJ (1992). The reviewing of object files: Object-specific integration of information. Cogn. Psychol..

[43] Van der Hallen R (2018). Connection-based and object-based grouping in multiple-object tracking: A developmental study. Br. J. Dev. Psychol..

[44] Awh E, Pashler H (2000). Evidence for split attentional foci. J. Exp. Psychol. Hum. Percept. Perform..

[45] McMains SA, Somers DC (2004). Multiple spotlights of attentional selection in human visual cortex. Neuron.

[46] Cavanagh P, Alvarez GA (2005). Tracking multiple targets with multifocal attention. Trends Cogn. Sci..

[47] Blankenship TL, Strong RW, Kibbe MM (2020). Development of multiple object tracking via multifocal attention. Dev. Psychol..

[48] Bowers, A. *et al*. *Dynamic Attention as a Predictor of Driving Performance in Clinical Populations: Preliminary Results*. 10.17077/drivingassessment.1413 (2011).

[49] Kelemen O (2007). How well do patients with schizophrenia track multiple moving targets?. Neuropsychology.

[50] Koldewyn K (2013). Multiple object tracking in autism spectrum disorders. J. Autism Dev. Disord..

[51] Ben-David BM, Nguyen LLT, van Lieshout PHHM (2011). Stroop effects in persons with traumatic brain injury: Selective attention, speed of processing, or color-naming? A meta-analysis. J. Int. Neuropsychol. Soc. JINS.

[52] Alnawmasi MM, Mani R, Khuu SK (2022). Changes in the components of visual attention following traumatic brain injury: A systematic review and meta-analysis. PLoS ONE.

[53] Cowan N (2001). The magical number 4 in short-term memory: A reconsideration of mental storage capacity. Behav. Brain Sci..

[54] Arciniega H (2019). Visual working memory deficits in undergraduates with a history of mild traumatic brain injury. Atten. Percept. Psychophys..

[55] Arciniega H (2021). Impaired visual working memory and reduced connectivity in undergraduates with a history of mild traumatic brain injury. Sci. Rep..

[56] Meyerhoff HS (2017). Studying visual attention using the multiple object tracking paradigm: A tutorial review. Atten. Percept. Psychophys..

[57] Meyerhoff HS, Papenmeier F (2020). Individual differences in visual attention: A short, reliable, open-source, and multilingual test of multiple object tracking in PsychoPy. Behav. Res. Methods Instrum. Comput..

[58] Alnæs D (2014). Pupil size signals mental effort deployed during multiple object tracking and predicts brain activity in the dorsal attention network and the locus coeruleus. J. Vis..

[59] Tullo D, Faubert J, Bertone A (2018). The characterization of attention resource capacity and its relationship with fluid reasoning intelligence: A multiple object tracking study. Intelligence.

[60] Willmott C (2009). Factors contributing to attentional impairments after traumatic brain injury. Neuropsychology.

[61] Ziino C, Ponsford J (2006). Selective attention deficits and subjective fatigue following traumatic brain injury. Neuropsychology.

[62] Hartikainen KM (2010). Persistent symptoms in mild to moderate traumatic brain injury associated with executive dysfunction. J. Clin. Exp. Neuropsychol..

[63] Arcia E, Gualtieri CT (1993). Association between patient report of symptoms after mild head injury and neurobehavioural performance. Brain Inj..

[64] Spencer RJ, Drag LL, Walker SJ (2010). Self-reported cognitive symptoms following mild traumatic brain injury are poorly associated with neuropsychological performance in OIF/OEF veterans. J. Rehabil. Res. Dev..

[65] McCrory P (2017). Consensus statement on concussion in sport—The 5th international conference on concussion in sport held in Berlin, October 2016. Br. J. Sports Med..

[66] McClincy MP (2006). Recovery from sports concussion in high school and collegiate athletes. Brain Inj..

[67] Hiploylee C (2017). Longitudinal study of postconcussion syndrome: not everyone recovers. J. Neurotrauma.

[68] Heitger MH (2006). Motor deficits and recovery during the first year following mild closed head injury. Brain Inj..

[69] Bernstein JP, Calamia M (2018). Assessing the longer-term effects of mild traumatic brain injury on self-reported driving ability. PM&R.

[70] Inamasu J (2018). Frequency and characteristics of traumatic brain injury in restrained drivers involved in road traffic accidents. Acta Neurochir..

[71] King N (1995). The Rivermead post concussion symptoms questionnaire: A measure of symptoms commonly experienced after head injury and its reliability. J. Neurol. Neurosurg. Psychiatry.

[72] Laukkanen H, Scheiman M, Hayes JR (2017). Brain injury vision symptom survey (BIVSS) questionnaire. Optom. Vis. Sci..

[73] Kessler RC (2005). The World Health Organization Adult ADHD Self-report Scale (ASRS): A short screening scale for use in the general population. Psychol. Med..

[74] Wechsler, D.J.T.P. *Wechsler Abbreviated Scale of Intelligence–Second Edition (WASI-II) San Antonio*. (2011).

[75] Wesemann W (2002). Visual acuity measured via the Freiburg visual acuity test (FVT), Bailey Lovie chart and Landolt Ring chart. Klin. Monatsbl. Augenheilkd..

[76] Trobe JD (1981). Confrontation visual field techniques in the detection of anterior visual pathway lesions. Ann. Neurol..

[77] Scheiman M, Wick B (2008). Clinical Management of Binocular Vision: Heterophoric, Accommodative, and Eye Movement Disorders.

[78] Grosvenor T, Grosvenor TP (2007). Primary Care Optometry.

[79] Mathworks, C. *User’s Guide R 2018 b*. (2018).

[80] Brainard DH (1997). The psychophysics toolbox. Vision..

[81] Prins N (2018). Applying the model-comparison approach to test specific research hypotheses in psychophysical research using the Palamedes toolbox. Front. Psychol. Rev..

[82] Brown GS, White KG (2005). The optimal correction for estimating extreme discriminability. Behav. Res. Methods Instrum. Comput..

[83] Cohen J (1992). A power primer. Psychol. Bull..

